# Measuring job stress of dental workers in China during the COVID-19 pandemic: reliability and validity of the hospital consultants’ job stress questionnaire

**DOI:** 10.1186/s12888-024-05670-x

**Published:** 2024-04-02

**Authors:** Huiqing Long, Li Yan, Xiaogang Zhong, Lu Yang, Yiyun Liu, Juncai Pu, Fangzhi Lou, Shihong Luo, Yingying Zhang, Yang Liu, Ping Ji, Xin Jin

**Affiliations:** 1 College of Stomatology, Chongqing Medical University, 401147 Chongqing, China; 2 Chongqing Key Laboratory of Oral Diseases and Biomedical Sciences, 401147 Chongqing, China; 3 Tongji Shanxi Hospital, Shanxi Bethune Hospital, Shanxi Academy of Medical Sciences, Third Hospital of Shanxi Medical University, 030032 Taiyuan, China; 4 College of Medical Informatics, Chongqing Medical University, 400016 Chongqing, China; 5 NHC Key Laboratory of Diagnosis and Treatment on Brain Functional Diseases, The First Affiliated Hospital of Chongqing Medical University, 400016 Chongqing, China

**Keywords:** Job stress, Reliability, Validity, Dental workers, China

## Abstract

**Background:**

The Hospital Consultants’ Job Stress Questionnaire (HCJSQ) has been widely used to assess sources and levels of job stress. However, its reliability and validity among Chinese dental workers have not been extensively studied. The objective of this study was to assess the reliability and validity of the HCJSQ specifically in Chinese dental workers.

**Methods:**

The HCJSQ was used to explore the sources and the global ratings of job stress among Chinese dental workers. To assess the reliability and validity of the HCJSQ, various statistical measures were employed, including Cronbach’s alpha coefficient, Spearman-Brown coefficient, Spearman correlation coefficient, exploratory factor analysis, confirmatory factor analysis, convergent validity, and discriminant validity.

**Results:**

Of the participants, 526 (17.4%) reported high levels of stress, while 1,246 (41.3%) and 1,248 (41.3%) reported moderate and low levels of stress, respectively. The Cronbach’s alpha coefficient for the modified HCJSQ was 0.903, and the Spearman-Brown coefficient was 0.904. Spearman correlation coefficient between individuals’ items and the total score ranged from 0.438 to 0.785 (*p* < 0.05). Exploratory factor analysis revealed that three factors accounted for 60.243% of the total variance. Confirmatory factor analysis demonstrated factor loadings between 0.624 and 0.834 on the specified items. The fit indices of the confirmatory factor analysis indicated good model fit, with a Root Mean Square Error of Approximation of 0.064, Normative Fit Index of 0.937, Comparative Fit Index of 0.952, Incremental Fit Index of 0.952, Tucker-Lewis index of 0.941, and Goodness of Fit Index of 0.944. Additionally, the convergent validity and discriminant validity showed a good fit for the three-factor model.

**Conclusion:**

The results of this study confirm that Chinese dental workers experience high levels of stress, and the three-factor model of the HCJSQ proves to be a suitable instrument for evaluating the sources and levels of job stress among Chinese dental workers. Therefore, it is imperative that relevant entities such as hospitals, medical associations, and government take appropriate measures to address the existing situation.

**Supplementary Information:**

The online version contains supplementary material available at 10.1186/s12888-024-05670-x.

## Introduction

Stress can be defined as a physiological and psychological response to a perceived threat, often experienced in the workplace when an individual feels that the demands of a situation outweigh their available resources and abilities [1]. Medical workers, such as trained and qualified physicians and nurses, are responsible for providing independent medical services to patients, as well as conducting supervision, training and management tasks within the healthcare industry. Consequently, these medical workers face a higher risk of stress and its associated consequences compared to the general population [2, 3]. This phenomenon is particularly prevalent in the field of dentistry, where the scarcity of dental workers in China has contributed to high job stress among dentists. According to the World Health Organization (WHO), the recommended dentist-to-population ratio is 1:5000, which increase to 1:2000 in developed countries. However, the current ratio of dental workers to population in China is less than 1:8000, significantly below the WHO’s recommended standard [4]. Moreover, Chinese medical workers, especially dental workers, also experience job stress due to high expectations from patients, intense competition among peers, occupational diseases such as lower back and cervical spine issues caused by heavy workloads and prolonged work hours, as well as strained relationships with colleagues [5, 6]. Especially, under the existing fierce Chinese medical title evaluation rules, dental workers are required not only to have skilled clinical skills, but also to master the scientific research ability to apply for scientific research projects and publish scientific research papers [7, 8]. Importantly, high job pressure not only negatively impacts the physical and mental well-being of dental workers but also hampers the rapid development of the social healthcare industry [9, 10]. Given the complex sources of job stress among dental workers and its adverse effects on the healthcare industry, it is essential to conduct a comprehensive assessment of their work-related stress levels to facilitate necessary interventions.

The COVID-19 pandemic has significantly intensified the challenges within the medical sector, notably in dentistry. Dental workers are particularly exposed to highly pathogenic environment for a long time [11, 12], such as aerosols containing a large number of pathogenic pathogens during the dental diagnosis and treatment processes [13, 14]. Additionally, the necessity of wearing cumbersome gear during the pandemic has introduced further complexities. These factors have notably escalated the stress levels among dental workers. The 19-Item Job Stress Scale was used to investigate the job stress of dental hygienists in South Korea, indicating a direct correlation between job stress and burnout among dental hygienists during the COVID-19 pandemic [15]. Additionally, the 10-item Perceived Stress Scale (PSS-10) was applied to assess the stress of dental staff in China during the spread of COVID-19, and it was found that the epidemic has placed greater pressure on dental workers, indicating that the incidence of mental symptoms was higher for front-line dental workers in oral emergency [16]. The reason is that during the epidemic, dental doctors and nurses often engaged in throat swab testing on the front line, which makes dental workers face the challenges such as excessive work burden and high risk of viral infection [17, 18]. In a word, the COVID-19 pandemic has adversely affected the working conditions and mental well-being of dental workers. However, the quality of scales used to measure the job stress of dental workers in the existing studies varied greatly, and the number of research subjects was limited. Therefore, there is an urgent need for suitable scales to analyze a wide range of Chinese dental workers.

It is exhilarating that there are many methods to assess the level of job stress among medical workers. However, some stress scales contain only one item without considering that the concept of job stress consists of multiple dimensions [19], and some stress scales are universal and not applicable to medical workers [20]. The Hospital Consultants’ Job Stress Questionnaire (HCJSQ), crafted by scholar Teasdale, is a 25-item self-report questionnaire designed to assess the level and sources of job stress among medical workers [21]. The accuracy and generality of the HCJSQ scale make it well-suited for assessing the stress levels of dental workers during the COVID-19 pandemic. Its comprehensive design allows for an in-depth exploration of the specific categories and underlying causes of increased stress in this challenging period. Furthermore, the scale takes into account the unique and complex work environment of medical professionals, and incorporates the specific sources of job stress experienced by medical workers. At present, the HCJSQ has been translated into various versions and extensively used in multiple regions, including India, Egypt, the United Kingdom, the Netherlands, and China [5, 22–25]. Nonetheless, most of the applications of HCJSQ were simply copied from the original version, without any evaluation of its reliability and validity. Additionally, there are no reports concerning the reliability and validity of HCJSQ among Chinese dental workers. Therefore, the objective of this study was to assess the reliability and validity of the HCJSQ specifically in Chinese dental workers. This will provide an effective tool for comprehending and screening the job stress experienced by Chinese dental workers following the outbreak of COVID-19.

## Materials and methods

### Participants

The data for this study were obtained from the first occupational survey of dental workers in China, carried out by the Chongqing Stomatological Association from February 2021 to March 2021. To select the sample, Chongqing Stomatological Association adopted the convenient sampling method, and selected medical institutions with stomatology departments (such as stomatological hospitals, stomatology department of general hospitals and dental clinics) in Southwest China as the research objects. Questionnaires were distributed to participants through an online survey platform named SoJump. First of all, Chongqing Stomatological Association contacted the directors of relevant hospitals/departments, who invited their employees to participate in this survey through the online platform of WeChat. Prior studies have recommended that the sample size for research should be at least 5 to 10 times larger than the number of scale items [26, 27]. At the same time, we calculated the sample size by using the following single population proportion formula: n = Z^2^×P×(1-P)/e^2^. In this formula, n = sample size; Z = confidence interval (1.96); *P* = prevalence (0.5); e = margin of sampling error to be tolerated (0.05) [28]. Therefore, the minimum sample size for this study was calculated as follows, with a confidence interval of 95% and margin of error 5% [*n* = 1.96^2^ × 0.5 × (1-0.5)/0.05^2^=384], plus a 10% non-response rate, resulting in a final calculation of 423. To minimize the risk of losing sample information, only fully completed questionnaires were accepted for submitted. If any unanswered questions were identified, participants were reminded to complete them. In order to ensure the accuracy of this questionnaire survey, if two or more consecutive questionnaires from the same hospital had identical answers, only one questionnaire was included, and the other identical questionnaires were considered invalid. In addition, if the same answer was chosen for the whole questionnaire items, the questionnaire was considered invalid.

### Research instruments

Each participant in the study received a self-administrated questionnaire consisting of two parts. The first part focused on demographic variables of dental workers, including gender, age, academic degree, technician status, monthly income, working years, weekly working hours, relationship status, parental status, number of patients treated per day, presence of a tube bed, type of hospital, job type, major, commuting time, and whether they undertake teaching tasks. The second part of the questionnaire was the self-administrated HCJSQ, which aimed to assess the sources and levels of job stress experienced by the participants [29]. The HCJSQ consisted of a 25-item self-reported questionnaire. Participants were asked to rate their perceived stress level over the past few months on a 4-pointed Likert-like scales, ranging from 0 to 3). The response options were “not at all”, “a little”, “quite a bit” and “a lot” of stress. Additionally, the Consultants’ Mental Health questionnaire was utilized to evaluate the overall ratings of job stress. It included a single question: “Overall, how stressful do you find your work?” Participants responded on a scale of 0–4. A score of ≥ 3 indicated high levels of overall stress, while a score of ≤ 1 indicated low levels of overall stress [30]. The questionnaire in detail was shown in Supplementary File 1.

### Statistical analysis

Data entry and verification were performed using Excel 2013, and all statistical analyses were conducted using SPSS 25.0 and AMOS 24.0. Mean and standard deviation were calculated for qualitative data. Scale reliability was evaluated using Cronbach’s α coefficient, Spearman-Brown coefficient, and coefficients above 0.70 were considered acceptable [31]. Additionally, Spearman’s correlation coefficient was used to assess reliability, with values above 0.30 considered acceptable [32]. To analyze the construct structure of HCJSQ, the sample was divided randomly, with three-quarters (2,265) used for exploratory factor analysis (EFA) and the remaining one quarter (755) for confirmatory analysis (CFA). EFA was conducted using principal component analysis and the maximum variance extraction method. Specific indicators were employed to evaluate the model fit. For the EFA analysis, criteria included a Kaiser-Meyer-Olkin (KMO) coefficient greater than 0.70, a significance level below 0.05 for Bartlett’s sphericity test, eigenvalues above 1.0, cumulative variance contribution rates above 50%, and factor loading above 0.40 [33, 34]. A cross-loading value below 0.30, and a difference of primary loading as compared to any cross-loading value above 0.2 were recommended [35]. CFA was assessed using the following model fit indices: (1) the Root Mean Square Error of Approximation (RMSEA) should be less than 0.10 (even better if below 0.05) to support model acceptance [36]. (2) Comparative Fit Index (CFI), Normative Fit Index (NFI), Incremental Fit Index (IFI), and Goodness of Fit Index (GFI) should be greater than 0.90 (preferably above 0.95) to indicate an appropriate fit [37, 38]. (3) ${\chi }^{2}/df$ was excluded, as it is highly sensitive to the sample size over 200 [39]. (4) Standardized factor loadings should be above 0.5 [40]. Convergent validity was assessed using the average variance extracted (AVE) and composite reliability (CR). AVE values above 0.5 and CR values above 0.7 indicated satisfactory convergent validity [41]. The discriminant validity of the constructs in the study was evaluated based on three criteria. Firstly, the inter-correlation values between the constructs should be lower than the square root of the AVE. Secondly, the AVE value of each construct should exceed its corresponding maximum shared variance (MSV). Lastly, the AVE value of each construct should be greater than its average shared variance (ASV) [42]. Statistical significance was determined by a *p*-value of less than 0.05.

## Results

### The demographic characteristics

As shown in the Table 1, the sample included 3,128 questionnaires from 11 provinces in China. After excluding 108 invalid questionnaires, there were 3,020 valid questionnaires, resulting in an effective rate of 96.55%. The number and reasons for excluding the questionnaires were presented in Supplementary Table 1. Among the respondents, the majority were female (*N* = 2,299, 76.1%), aged 20–35 years old (*N* = 1,984, 65.7%), married (*N* = 2,022, 67.0%), worked < 10 years (*N* = 2,009, 66.5%), worked < 45 h per week (*N* = 2,172, 71.9%), not tube bed (*N* = 2,675, 88.65%), worked in a dental specialty hospital (*N* = 1,975, 65.4%), doctors (*N* = 1,855, 61.4%) and did not undertake teaching tasks (*N* = 2,010, 66.6%).

**Table 1:** The demographic characteristics of the participants

Items	N (%)	Items	N (%)
Gender		Whether have children	
Male	721 (23.9)	No	1,249 (41.4)
Female	2,299 (76.1)	Yes	1,771 (58.6)
Academic degree obtained		Treated patients per day	
Doctor’s degree	137 (4.5)	< 10	940 (31.1)
Master’s degree	740 (24.5)	10–20	1,285 (42.5)
Bachelor’s degree	1,659 (54.9)	21–30	415 (13.7)
College’s degree or below	484 (16.0)	> 30	380 (12.6)
Age (years)		Whether tube bed	
20–35	1,984 (65.7)	No	2,675 (88.6)
36–50	862 (28.5)	Yes	345 (11.4)
> 50	174 (5.8)	Hospital type	
Technician		Dental specialist hospital	1,975 (65.4)
Junior	1,763 (58.4)	General hospital	881 (29.2)
Intermediate	885 (29.3)	Private hospital	164 (5.4)
Senior	372 (12.3)	Job type	
Monthly income (RMB)		Doctor	1,855 (61.4)
< 5,000	842 (27.9)	Nurse	1,165 (38.6)
5,000–10,000	1,372 (45.4)	Major type	
10,000–15,000	415 (13.7)	General	1,331 (44.1)
> 15,000	391 (12.9)	Internal medicine	662 (21.9)
Working years		Maxillofacial surgery	339 (11.2)
< 10	2,009 (66.5)	Prosthodontics	269 (8.9)
10–20	636 (21.1)	Implant	114 (3.8)
> 20	375 (12.4)	Orthodontics	305 (10.1)
Hours worked per week		Commuting time (minutes)	
< 45	2,172 (71.9)	< 15	544 (18.0)
45–55	639 (21.1)	15–30	1,235 (40.9)
> 55	209 (6.9)	31–45	615 (20.4)
Relationship status		46–60	397 (13.1)
Single	565 (18.8)	> 60	229 (7.6)
Partnered	359 (11.9)	Whether undertake teaching tasks	
Married	2,022 (67.0)	Yes	1,010 (33.4)
Divorced or widowed	72 (2.4)	No	2,010 (66.6)

### The characteristics of the overall job stress and HCJSQ

Among the 3,020 dental workers who participated in this survey, the number of individuals experiencing low, moderate and high levels of job stress was 1,248 (41.3%), 1,246 (41.3%), and 526 (17.4%), respectively.

To assess the relative importance of different sources of stress, we calculated the percentage of medical workers who reported each item as contributing “quite a bit” or “a lot” to their job stress [21]. The results revealed the top five sources of job stress among dental workers: (1) Feeling you are poorly paid for the job you do (1,244/3,020, 41.2%); (2) Keeping up to date with current clinical and research practices (1,083/3,020, 35.9%); (3) Being responsible for the quality of the work of other staff (901/3,020, 29.8%); (4) Feeling the medical workers in the department is inadequate (814/3,020, 26.9%); (5) Having conflicting demands on your time (e.g. patient care/management/research/College) (784/3,020, 25.9%). The details were shown in Table 2.

**Table 2:** Item responses of HCJSQ (*n* = 3,020)

Items	Item respond number and rate (N/%)			
	Not at all	A little	Quite A bit	A Lot
1. Being involved with the physical suffering of patients	989 (32.7)	1438 (47.6)	517 (17.1)	76 (2.5)
2. Encountering difficulties in relationships with junior medical staff	2409 (79.8)	481 (15.9)	112 (3.7)	18 (0.6)
3. Feeling you have insufficient input into the management of your unit or institution	1771 (58.6)	958 (31.7)	246 (8.1)	45 (1.5)
4. Disruption of your home life through spending long hours at work	1208 (40.0)	1191 (39.4)	486 (16.1)	135 (4.5)
5. Having inadequate facilities (e.g. equipment, space) to do your job properly	1165 (38.6)	1194 (39.5)	498 (16.5)	163 (5.4)
6. Having to deal with distressed, angry or blaming relatives	1037 (34.3)	1389 (46)	496 (16.4)	98 (3.2)
7. Keeping up to date with current clinical and research practices	733 (24.3)	1204 (39.9)	847 (28.0)	236 (7.8)
8. Having to take on more managerial responsibilities	1191 (39.4)	1236 (40.9)	462 (15.3)	131 (4.3)
9. Encountering difficulties in relationships with consultant colleagues	2357 (78.0)	541 (17.9)	108 (3.6)	14 (0.5)
10. Feeling under pressure to meet deadlines	996 (33.0)	1378 (45.6)	533 (17.6)	113 (3.7)
11. Being responsible for the quality of the work of other staff	968 (32.1)	1151 (38.1)	619 (20.5)	282 (9.3)
12. Being involved with the emotional distress of patients	1102 (36.5)	1524 (50.5)	332 (11)	62 (2.1)
13. Encountering difficulties in relationships with administrative staff, e.g. secretaries	1958 (64.8)	825 (27.3)	194 (6.4)	43 (1.4)
14. Having too great an overall volume of work	988 (32.7)	1370 (45.4)	532 (17.6)	130 (4.3)
15. Feeling you are poorly paid for the job you do	724 (24.0)	1052 (34.8)	814 (27.0)	430 (14.2)
16. Encountering difficulties in relationships with managers	1691 (56.0)	1013 (33.5)	237 (7.8)	79 (2.6)
17. Having conflicting demands on your time (e.g. patient care/management/research/College)	1006 (33.3)	1230 (40.7)	567 (18.8)	217 (7.2)
18. Feeling the medical workers in the department is inadequate	1138 (37.7)	1068 (35.4)	555 (18.4)	259 (8.6)
19. Worried about being complained or sued for improper treatment of patients	1074 (35.6)	1272 (42.1)	488 (16.2)	186 (6.2)
20. Disruption of your home life as a result of taking paperwork Home (e.g. research practice)	1221 (40.4)	1210 (40.1)	445 (14.7)	144 (4.8)
21. Feeling that your accumulated skills and expertise are not being put to their best use	1216 (40.3)	1274 (42.2)	417 (13.8)	113 (3.7)
22. Disruption of your home life as a result of being on duty	1366 (45.2)	1125 (37.3)	376 (12.5)	153 (5.1)
23. Having a conflict of responsibilities (e.g. clinical vs. managerial; clinical vs. research)	1411 (46.7)	1091 (36.1)	377 (12.5)	141 (4.7)
24. Uncertainty over the future development of your unit/institution	1361 (45.1)	1056 (35.0)	450 (14.9)	153 (5.1)
25. Being responsible for the welfare of other staff	1425 (47.2)	985 (32.6)	433 (14.3)	177 (5.9)

Abbreviations: HCJSQ: The Hospital Consultants’ Job Stress Questionnaire

### Exploratory factor analysis

The HCJSQ was tested in a sample of 2,265 participants. Initially, the KMO measure and Bartlett’s sphericity test were conducted on 25 items. The results indicated a KMO coefficient of 0.957 and an approximate chi-square value of 30,332 (*df* = 300, *p* < 0.001), suggesting that the scale was suitable for factor analysis. To carry out the exploratory factor analysis using principal component analysis and the maximum variance rotation method, items 3, 6, 10, 13 and 17 were deleted due to cross-loading > 0.3. Additionally, items 1, 8, 12, 16 and 19 were deleted due to differences between primary loading and cross-loading value < 0.2. After removing these items, the final exploratory factor analysis was performed. The results of the factor analysis showed a KMO coefficient of 0.924 and an approximate chi-square value of 15,065 (*df* = 105, *p* < 0.001). Three common factors with eigenvalues > 1 were extracted, as shown in Supplementary Fig. 1. Common factor 1 included 10 items: item 4, 5, 14, 15, 18, 20, 21, 22, 23, and 24, with a variance contribution rate of 43.412%. Common factor 2 consisted of three items: item 7, 11, and 25, with a variance contribution rate of 8.568%. Common factor 3 comprised two items: item 2 and 9, with a variance contribution rate of 8.264%. The cumulative variance contribution rate of the three-factor model was 60.243%, surpassing the acceptable standard of 50% [43]. Additionally, the factor loadings ranged from 0.595 to 0.859 (Table 3).

**Table 3:** Factor load of the EFA for the modified HCJSQ (*n* = 2,265)

Items	Common factor 1	Common factor 2	Common factor 3
2. Encountering difficulties in relationships with junior medical staff	0.192	0.097	0.859
4. Disruption of your home life through spending long hours at work	0.704	0.161	0.206
5. Having inadequate facilities (e.g. equipment, space) to do your job properly	0.668	0.097	0.168
7. Keeping up to date with current clinical and research	0.212	0.677	0.02
9. Encountering difficulties in relationships with consultant colleagues	0.233	0.111	0.844
11. Being responsible for the quality of the work of other staff	0.170	0.83	0.055
14. Having too great an overall volume of work	0.760	0.254	0.102
15. Feeling you are poorly paid for the job you do	0.713	0.149	-0.105
18. Feeling the medical workers in the department is inadequate	0.595	0.262	0.063
20. Disruption of your home life as a result of taking paperwork Home (e.g. research practice)	0.708	0.233	0.168
21. Feeling that your accumulated skills and expertise are not being put to their best use	0.693	0.157	0.247
22. Disruption of your home life as a result of being on duty	0.742	0.102	0.214
23. Having a conflict of responsibilities (e.g. clinical vs. managerial; clinical vs. research)	0.689	0.289	0.242
24. Uncertainty over the future development of your unit/institution	0.723	0.147	0.200
25. Being responsible for the welfare of other staff	0.234	0.727	0.199
Variance contribution rate	43.412%	8.568%	8.264%
Cumulative variance contribution rate	43.412%	51.979%	60.243%

Abbreviations: EFA: exploratory factor analysis; HCJSQ: The Hospital Consultants’ Job Stress Questionnaire

### Confirmatory factor analysis

**Fig. 1:**
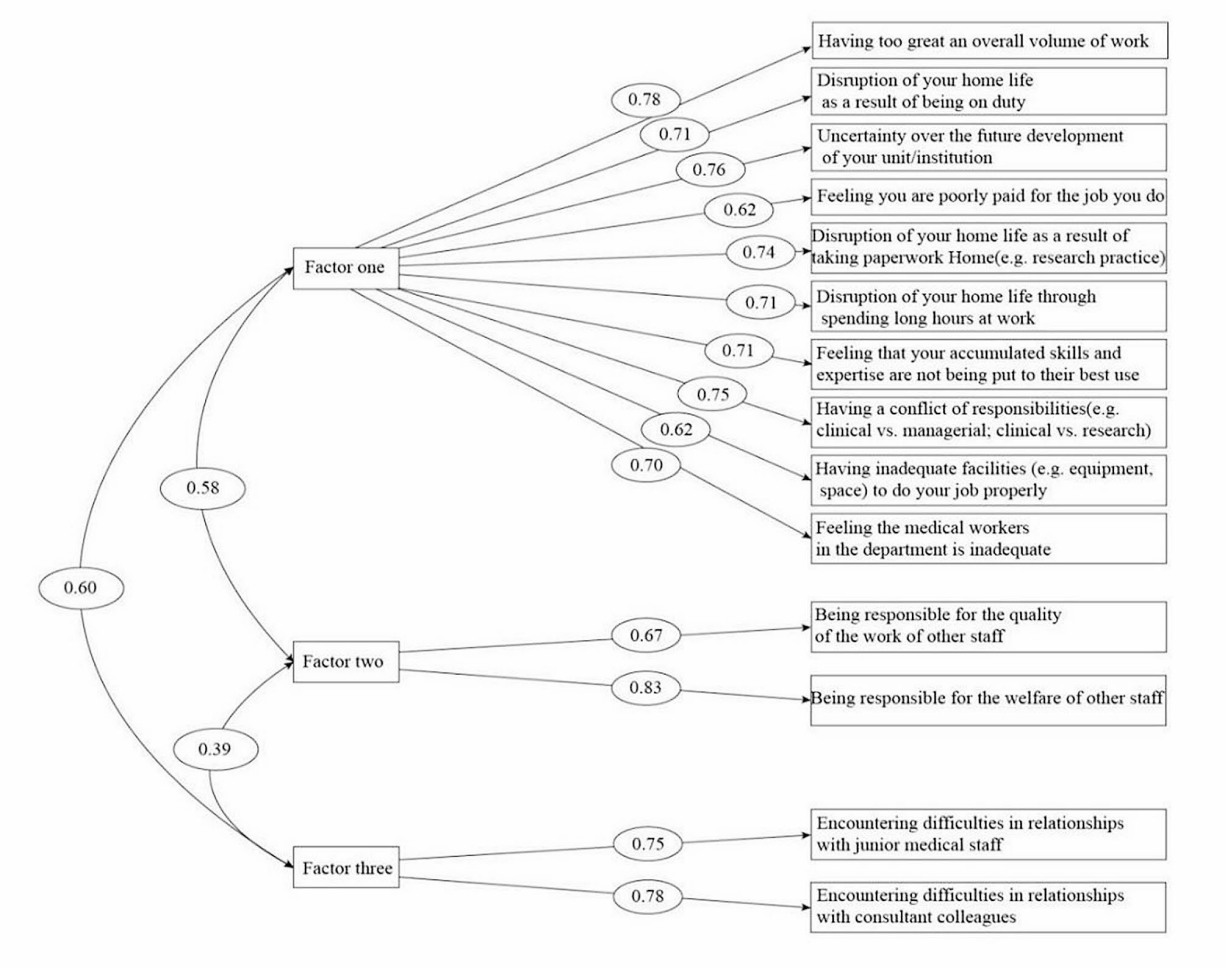
Standard factor load of three-factor model

To assess the suitability of the structure revealed by the EFA, the maximum likelihood method was utilized to conduct the CFA. During the CFA, item 7 was deleted because its standardized factor load was less than 0.50. The CFA was then re-performed, yielding a ${\chi }^{2}/df$ value of 4.112 and an RMSEA value of 0.064. The values for NFI, CFI, IFI, TLI, and GFI were 0.937, 0.952, 0.952, 0.941, and 0.944, respectively. The factor loadings on the specified factors for each item, as indicated by the AMOS path, ranged from 0.624 to 0.834 (Fig. 1). All of the aforementioned indices exceeded the recommended thresholds, suggesting that the validity of the three-factor model of the HCJSQ was acceptable.

### Convergent validity and discriminant validity

Furthermore, we also examined the convergent validity and discriminant validity of the three-factor model. Both the CR and AVE exceed the recommended values, demonstrating the three-factor model has convergent validity (MSV < AVE, and ASV < AVE). When the correlation coefficient between the two factors was less than the square root of the average variation, the divergence validity was also desirable (Table 4). The results showed that the three-factor model fit the data very well. Additionally, we also tested the four-factor model of the original scale in our data, and the results showed the three-factor model provided better fit than four-factor model (Supplementary Table 2).

**Table 4:** The convergent validity and discriminant validity of three-factor model

Common factor	CR	AVE	ASV	MSV	F1	F2	F3
Factor one	0.911	0.507	0.349	0.358	0.712		
Factor two	0.725	0.572	0.244	0.340	0.583	0.756	
Factor three	0.737	0.584	0.253	0.358	0.598	0.386	0.764

Abbreviations: CR: composite reliability; AVE: average variance extracted; ASV: average shared variance. F1: factor 1; F2: factor 2; F3: factor 3

### Reliability and correlations analysis

The reliability analysis revealed that the Cronbach’s α coefficient of the modified total scale was 0.903, and for the three common factors, the coefficients were 0.907, 0.692, and 0.755, respectively. After excluding the individual items, the Cronbach’s α coefficient for the total scale ranged from 0.892 to 0.904 (Table 5). The Spearman-Brown coefficient, indicating half-fold reliability, was 0.904, suggesting that the scale had acceptable reliability.

**Table 5:** The Cronbach’ s α and correlations coefficient for the modified HCJSQ (*n* = 3,020)

Items	Cronbach’s α after the item deleted	Correlation coefficient with total score
2. Encountering difficulties in relationships with junior medical staff	0.903	0.438
4. Disruption of your home life through spending long hours at work	0.894	0.708
5. Having inadequate facilities (e.g. equipment, space) to do your job properly	0.896	0.663
9. Encountering difficulties in relationships with consultant colleagues	0.902	0.488
11. Being responsible for the quality of the work of other staff	0.904	0.535
14. Having too great an overall volume of work	0.891	0.785
15. Feeling you are poorly paid for the job you do	0.898	0.632
18. Feeling the medical workers in the department is inadequate	0.897	0.648
20. Disruption of your home life as a result of taking paperwork home (e.g. research practice)	0.893	0.738
21. Feeling that your accumulated skills and expertise are not being put to their best use	0.893	0.720
22. Disruption of your home life as a result of being on duty	0.893	0.703
23. Having a conflict of responsibilities (e.g. clinical vs. managerial; clinical vs. research)	0.892	0.767
24. Uncertainty over the future development of your unit/institution	0.892	0.743
25. Being responsible for the welfare of other staff	0.901	0.579

Abbreviations: HCJSQ: The Hospital Consultants’ Job Stress Questionnaire

Spearman correlation analysis demonstrated that the correlation coefficients between item scores and total scores ranged from 0.438 to 0.785 (*p* < 0.05). This analysis revealed that item 2 had the lowest correlation, while item 14 had the highest correlation with the total scores. Furthermore, the correlation coefficients among the inter-item scores ranged from 0.155 to 0.656. It is worth noting that the correlation coefficients between items and the total scores were higher than those among the inter-items, indicating that the reliability of the scale was acceptable (Supplementary Table 3).

## Discussion

HCJSQ is one of the most widespread scales to measure job stress, and has been translated into various versions worldwide. However, there are few studies that have tested the validity of the scale, with most researchers referring solely on the original work done by the developers [5, 22–25]. Given the popularity of HCJSQ internationally and the lack of research on its psychometric properties of the scale, this study aimed to investigate the construct validity and internal consistency of HCJSQ for Chinese dental workers.

In this study, 17.4% of the respondents reported experiencing high levels of job stress (526/3020), which was lower than the rate reported by Egyptian physicians (37.8%) [23]. The low rate of high job stress among dental workers might be attributed to the effective prevention and control measures taken in response to the epidemic situation in China. In order to establish the validity of HCJSQ, EFA and CFA were employed to identify common factor and assess the model separately. EFA revealed the extraction of three common factors from HCJSQ, with eigenvalues greater than 1.0. The factor loadings were relatively high, ranging from 0.595 to 0.859. The cumulative variance contribution rate of the three-factor model accounted for 60.243% of the total variance observed in HCJSQ, surpassing the recommended threshold of 50.0% [36]. In the three-factor model, workload and work resources were found to be grouped together as factor one, indicating that these items can be associated with and explained by the same underlying construct. Factor two comprised stressors related to management responsibilities for other staff members, while factor three included stressors associated with interactions with colleagues. The above three factors basically include all aspects of job stress among dental workers from work ability, working environment and interpersonal relationship [10], which has multiple adverse effects on the quality of the work of dental workers. Several reasons could explain these findings: on the one hand, there was a strong correlation between the items within each factor [44]; on the other hand, some stressors were found to have more serious adverse consequences. For example, stress resulting from interpersonal conflicts could lead to more noticeable mood disorders, thus supporting the accuracy of convergent and discriminant validity of the three-factor model [45]. In addition, Chinese traditional culture places a high value on collectivism and interpersonal communication, leading to individuals experiencing job stress primarily through interpersonal relationship [46]. This is particularly true for individuals who identify with collectivist cultures, as they typically strive to avoid explicit interpersonal conflict in their interactions [47]. Moreover, the Cronbach’s α coefficient of the revised HCJSQ is 0.903, and the Spearman-Brown coefficient of the revised aggregate table is 0.904, which indicates that the reliability of the revised scale is appropriate. In summary, based on the reliability and validity analysis of the HCJSQ scale in this study, the modified three-factor model of the HCJSQ is proved to be a valid and reliable tool for measuring the job stress of Chinese dental workers.

Additionally, CFA is an important statistical method used to assess the reliability and validity of the measurement scales [48]. While CFA has been previously used to verify the psychometric properties of job stress scales in various professions, such as police and nurses [49, 50], there is no existing literature on the application of CFA to verify the established factor structure of the HCJSQ scale. Our CFA results showed that the RSMEA for the three-factor model was lower than the recommended value of 0.10, and the goodness-of-fit indices (NFI, IFI, TLI and GFI) were higher than the recommended value of 0.90. These findings suggest that our three-factor model effectively explains the level and sources of job stress experienced by Chinese dental workers. Furthermore, to establish the convergent and discriminant validity of the three-factor model, several tests were conducted. The results revealed that the AVE for each construct was greater than 0.5 and the CR was higher than 0.7, indicating satisfactory convergent validity. The inter-correlation values between the constructs were found to be lower than the square root of the AVE, demonstrating discriminant validity. Additionally, the AVE was greater than the MSV and the ASV, which further confirmed the three-factor model’s convergent and discriminant validity. It is worth noting that we also tested the four-factor model of the original scale in our dataset, and the results favored the three-factor model as a better fit for Chinese dental workers. This research strategy is similar to the previous reliability and validity analysis of other psychological scales, which used single-factor model, two-factor model, and even multi-factor model to fit the data with to make indicators meet its criterion [51, 52]. By considering these factors, we can conclude that our study provides robust evidence to support the advantages of using a three-factor model in assessing the level and sources of job stress among Chinese dental workers.

Previous studies have shown a link between job stress and various negative outcomes, including lower professional quality of life, job burnout, lower sleep quality, and depressive symptoms [53–55]. Interestingly, a report from the British Dental Association highlighted that dentists experience higher levels of stress compared to the general population [56]. This could be attributed to factors such as high career expectations and excessive workload [57]. Additionally, dental workers are often exposed to occupational hazards, such as aerosols containing pathogenic microbiota and contact with blood and body fluids of patients with infectious diseases. These factors partly explain the high levels of job stress reported among dental workers [58, 59]. Once the levels and stressors of job stress among dental workers have been identified, it is important to provide them with techniques to build resilience against job stress. Medical institutions should consider establishing special training and coaching programs for dentists to enhance their problem-solving and communication skills [60]. Dental workers themselves can also adopt measures to reduce job stress, including mindfulness training, massage therapy, and listening to soothing music [61, 62]. In addition, the government and relevant departments should take appropriate measures to alleviate job stress among dental workers. This could involve workload modification, improvement of shift hours, and increasing incomes to address the specific challenges faced by dental professionals and create a more supportive work environment. In short, the solution of the social problem of reducing the job stress of dental workers needs the joint efforts of individuals, work units and social departments.

### Limitations

There are also several limitations in this study. Firstly, this study utilized a convenience sampling method, which may introduce selection bias into the data. Secondly, this study did not establish a gold standard for measuring job stress, resulting in a lack of analysis regarding the validity of the efficacy standard. Thirdly, there may be potential bias in the self-report scale results of HCJSQ, and future studies could consider incorporating objective biomarkers such as cortisol levels. Fourthly, the reset reliability analysis was not performed in this study. Fifthly, it is recommended to employ the Chinese version of HCJSQ to evaluate the stress levels of dental workers during the post-pandemic era and other public emergencies. Additionally, the survey participants in this study were limited to dental workers, thus, it may not applicable to the general or other professional population. Given the aforementioned limitations, further research is necessary to corroborate our findings.

## Conclusions

The results of this study demonstrate that the HCJSQ is a reliable and valid tool for assessing job stressors and levels among dental workers in China during the COVID-19 pandemic. This study represents the first systematic investigation into the stress levels experienced by dental workers in southwest China during the COVID-19 pandemic, and the results show that Chinese dental workers experience high levels of stress, highlighting the need for hospitals, medical associations, and other relevant entities to take appropriate measures to address this issue.

### Electronic supplementary material

Below is the link to the electronic supplementary material.

Supplementary File 1: A survey of the job stress of Chinese dental medical workers. Supplementary Table 1: The number and reasons for the excluded questionnaires. Supplementary Table 2: The convergent validity and discriminant validity of four-factor model. Supplementary Table 3: Spearman correlation analysis for the revised HCJSQ scale. Supplementary Figure 1: Screen plot of the exploratory factor analysis.

## Data Availability

The data supporting the conclusions of this article are included within the article.
