# Supplementary material for: Measuring job stress of dental workers in China during the COVID-19 pandemic: reliability and validity of the hospital consultants’ job stress questionnaire
Source: BMC Psychiatry. 2024 Apr 2;24:246. doi: 10.1186/s12888-024-05670-x (PMC10985848; doi:10.1186/s12888-024-05670-x)
Supplement: Supplementary file 1 — A survey of the job stress of Chinese dental medical workers. Supplementary Table 1: The number and reasons for the excluded questionnaires. Supplementary Table 2: The convergent validity and discriminant validity of four-factor model. Supplementary Table 3: Spearman correlation analysis for the revised HCJSQ scale. Supplementary Figure 1: Screen plot of the exploratory factor analysis. [file 12888_2024_5670_MOESM1_ESM.docx]

**Supplementary File 1**

**A survey of the job stress of Chinese dental medical workers**

**Answer instructions**

1. Please tick "√" on the answer number suitable for your situation after each question, or fill in the appropriate content at____.

2. In the process of filling in the questionnaire, please do not discuss with others, and fill in according to your actual situation.

**Part Ⅰ. Basic personal information.**

Please fill in your true information and tick the corresponding brackets.

Full name of the hospital: _________________________.

**Q.1.** Gender:

Male ( )

Female ( )

**Q.2.** Academic degree obtained:

Doctor's degree ( )

Master's degree ( )

Bachelor's degree ( )

College's degree or below ( )

**Q.3.** Your age group:

20 - 35 years old ( )

36 - 50 years old ( )

Over 50 years old ( )

**Q.4.** Technical title:

Junior ( )

Intermediate ( )

Senior ( )

**Q.5.** Your monthly income:

< 5,000 RMB ( )

5,000 - 10000 RMB ( )

10,000-15,000 RMB ( )

> 15,000 RMB ( )

**Q.6.** Working years:

< 10 years ( )

10 - 20 years ( )

> 20 years ( )

**Q.7.** Hours worked per week:

< 45 hours ( )

45 - 55 hours ( )

> 55 hours ( )

**Q.8.** Relationship status:

Single ( )

Partnered ( )

Married ( )

Divorced or widowed ( )

**Q.9.** Whether have children:

NO ( )

Yes ( )

**Q.10.** Treated patients per day:

< 10 ( )

10 - 20 ( )

21 - 30 ( )

> 30 ( )

**Q.11.** Whether tube bed:

No ( )

Yes ( )

**Q.12.** Hospital type:

Dental specialist hospital ( )

General hospital ( )

Private hospital ( )

**Q.13.** Job type:

Doctor ( )

Nurse ( )

**Q.14.** Major type:

General ( )

Internal medicine ( )

Maxillofacial surgery ( )

Prosthodontics ( )

Implant ( )

Orthodontics ( )

**Q.15.** Commuting time(minutes):

< 15

15 - 30

31 -45

46 - 60

> 60

**Q.16.** Whether undertake teaching tasks:

Yes ( )

No ( )

**Part Ⅱ. Assessment the sources of current job stress and overall stress levels.**

1. In the past few months of work, how did the following projects put stress on you? Please indicate the stress level with 0, 1, 2 and 3, and tick the corresponding level items. If you do not feel stress, it is indicated with 0.

| **Items** | **Stress level** | | | |
| --- | --- | --- | --- | --- |
|  | **Not at all** | **A little** | **Quite A bit** | **A Lot** |
| **Q.1.** Being involved with the physical suffering of patients | 0 | 1 | 2 | 3 |
| **Q.2.** Encountering difficulties in relationships with junior medical staff | 0 | 1 | 2 | 3 |
| **Q.3.** Feeling you have insufficient input into the management of your unit or institution | 0 | 1 | 2 | 3 |
| **Q.4.** Disruption of your home life through spending long hours at work | 0 | 1 | 2 | 3 |
| **Q.5.** Having inadequate facilities (e.g. equipment, space) to do your job properly | 0 | 1 | 2 | 3 |
| **Q.6.** Having to deal with distressed, angry or blaming relatives | 0 | 1 | 2 | 3 |
| **Q.7.** Keeping up to date with current clinical and research practices | 0 | 1 | 2 | 3 |
| **Q.8.** Having to take on more managerial responsibilities | 0 | 1 | 2 | 3 |
| **Q.9.** Encountering difficulties in relationships with consultant colleagues | 0 | 1 | 2 | 3 |
| **Q.10.** Feeling under pressure to meet deadlines | 0 | 1 | 2 | 3 |
| **Q.11.** Being responsible for the quality of the work of other staff | 0 | 1 | 2 | 3 |
| **Q.12.** Being involved with the emotional distress of patients | 0 | 1 | 2 | 3 |
| **Q.13.** Encountering difficulties in relationships with administrative staff, e.g. secretaries | 0 | 1 | 2 | 3 |
| **Q.14.** Having too great an overall volume of work | 0 | 1 | 2 | 3 |
| **Q.15.** Feeling you are poorly paid for the job you do | 0 | 1 | 2 | 3 |
| **Q.16.** Encountering difficulties in relationships with managers | 0 | 1 | 2 | 3 |
| **Q.17.** Having conflicting demands on your time (e.g. patient care/management/research/College) | 0 | 1 | 2 | 3 |
| **Q.18.** Feeling the medical workers in the department is inadequate | 0 | 1 | 2 | 3 |
| **Q.19.** Worried about being complained or sued for improper treatment of patients | 0 | 1 | 2 | 3 |
| **Q.20.** Disruption of your home life as a result of taking paperwork Home (e.g. research practice) | 0 | 1 | 2 | 3 |
| **Q.21.** Feeling that your accumulated skills and expertise are not being put to their best use | 0 | 1 | 2 | 3 |
| **Q.22.** Disruption of your home life as a result of being on duty | 0 | 1 | 2 | 3 |
| **Q.23.** Having a conflict of responsibilities (e.g. clinical vs. managerial; clinical vs. research) | 0 | 1 | 2 | 3 |
| **Q.24.** Uncertainty over the future development of your unit/institution | 0 | 1 | 2 | 3 |
| **Q.25.** Being responsible for the welfare of other staff | 0 | 1 | 2 | 3 |

1. Overall, how stressful do you find your work? Please indicate the stress level with 0, 1, 2, 3, and 4, and tick the corresponding level items.

| **Items** | **Stress level** | | | | |
| --- | --- | --- | --- | --- | --- |
| **Q.1.** Overall, how stressful do you find your work? | 0 | 1 | 2 | 3 | 4 |

**Supplementary Table 1:** The number and reasons for the excluded questionnaires.

| **Number** | **Reasons** |
| --- | --- |
| 99 | The answers of the whole questionnaire items were same |
| 9 | The answers of questionnaires from the same hospital were identical |

**Supplementary Table 2:** The convergent validity and discriminant validity of four-factor model.

| Common factor | CR | AVE | ASV | MSV | F1 | F2 | F3 | F4 |
| --- | --- | --- | --- | --- | --- | --- | --- | --- |
| Factor one | 0.900 | 0.602 | 0.677 | 0.758 | 0.776 |  |  |  |
| Factor two | 0.851 | 0.489 | 0.604 | 0.758 | 0.871 | 0.699 |  |  |
| Factor three | 0.729 | 0.576 | 0.48 | 0.521 | 0.722 | 0.678 | 0.759 |  |
| Factor four | 0.601 | 0.434 | 0.6 | 0.749 | 0.865 | 0.770 | 0.677 | 0.659 |

**Supplementary Table 3:** Spearman correlation analysis for the revised HCJSQ scale.

| Items | Item 2 | Item 4 | Item 5 | Item 9 | Item 11 | Item 14 | Item 15 | Item 18 | Item 20 | Item 21 | Item 22 | Item 23 | Item 24 | Item 25 | Total score |
| --- | --- | --- | --- | --- | --- | --- | --- | --- | --- | --- | --- | --- | --- | --- | --- |
| Item 2 | 1 |  |  |  |  |  |  |  |  |  |  |  |  |  |  |
| Item 4 | .328* | 1 |  |  |  |  |  |  |  |  |  |  |  |  |  |
| Item 5 | .285* | .478* | 1 |  |  |  |  |  |  |  |  |  |  |  |  |
| Item 9 | .567* | .335* | .324* | 1 |  |  |  |  |  |  |  |  |  |  |  |
| Item 11 | .177* | .298* | .266* | .203* | 1 |  |  |  |  |  |  |  |  |  |  |
| Item 14 | .281* | .626* | .475* | .309* | .383* | 1 |  |  |  |  |  |  |  |  |  |
| Item 15 | .155* | .411* | .429* | .175* | .297* | .552* | 1 |  |  |  |  |  |  |  |  |
| Item 18 | .263* | .435* | .446* | .281* | .330* | .508* | .427* | 1 |  |  |  |  |  |  |  |
| Item 20 | .303* | .587* | .435* | .341* | .317* | .589* | .412* | .455* | 1 |  |  |  |  |  |  |
| Item 21 | .328* | .459* | .535* | .377* | .297* | .535* | .478* | .456* | .531* | 1 |  |  |  |  |  |
| Item 22 | .340* | .592* | .450* | .379* | .282* | .554* | .439* | .460* | .592* | .526* | 1 |  |  |  |  |
| Item 23 | .340* | .515* | .463* | .396* | .346* | .591* | .426* | .462* | .656* | .552* | .611* | 1 |  |  |  |
| Item 24 | .335* | .465* | .546* | .379* | .292* | .538* | .495* | .472* | .511* | .621* | .542* | .614* | 1 |  |  |
| Item 25 | .268* | .350* | .311* | .306* | .535* | .395* | .253* | .378* | .388* | .392* | .375* | .458* | .424* | 1 |  |
| Total score | .438* | .708* | .663* | .488* | .535* | .785* | .632* | .648* | .738* | .720* | .703* | .767* | .743* | .579* | 1 |
| * At 0.05 level (two tailed), the correlation was significant. | | | | | | | | | | | | | | | |


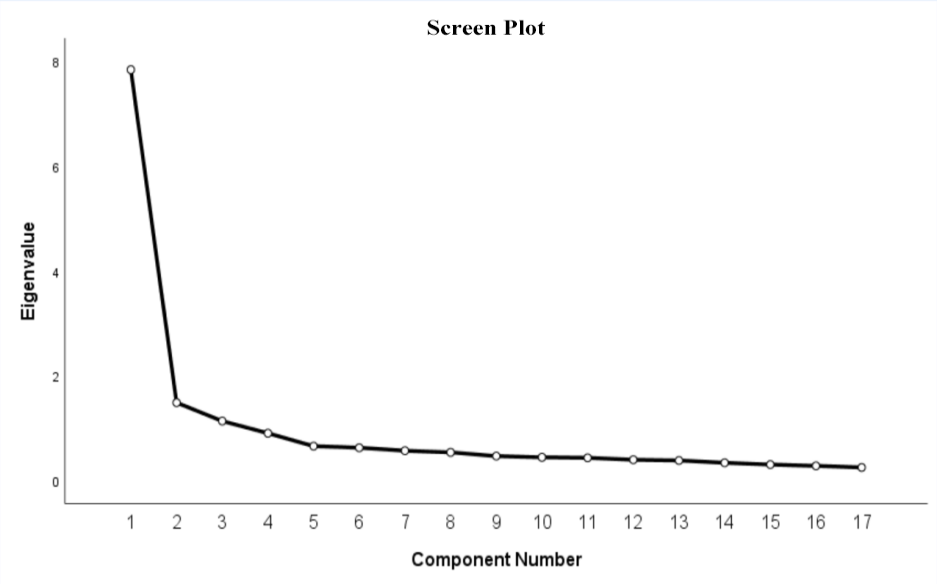


**Supplementary Figure 1:** Screen plot of the exploratory factor analysis.
